# Boredom Intervention Training Phase I: Increasing Boredom Knowledge through a Psychoeducational Video

**DOI:** 10.3390/ijerph182111712

**Published:** 2021-11-08

**Authors:** Patti C. Parker, Virginia M. C. Tze, Lia M. Daniels, Alyse Sukovieff

**Affiliations:** 1 Department of Educational Psychology, Faculty of Education, University of Alberta, Edmonton, AB T6G 2G5, Canada; pparker@ualberta.ca (P.C.P.); lia1@ualberta.ca (L.M.D.); 2 Department of Educational Administration, Foundations & Psychology, Faculty of Education, University of Manitoba, Winnipeg, MB R3T 2N2, Canada; 3 Department of Psychology, Faculty of Arts, University of Manitoba, Winnipeg, MB R3T 2N2, Canada; newmana3@myumanitoba.ca

**Keywords:** boredom, achievement emotions, psychoeducation, intervention, control-value theory, component process model of emotions

## Abstract

Boredom is a salient emotion experienced in postsecondary settings, and evidence reveals that it can negatively impact academic achievement and motivation. Drawing from the control-value theory (CVT) of achievement emotions (Pekrun, 2006) and the component process model of emotions (CPM; Scherer, 1984), our study examines the first phase of a multi-sequenced online boredom intervention training (BIT) program. The goal of Phase I of BIT was to increase university students’ (*N* = 85) knowledge about boredom as a scholarly construct. Students completed four components of the Phase I BIT session, including: (a) a baseline survey and knowledge quiz, (b) a psychoeducational video, (c) a consolidation exercise, and (d) a follow-up knowledge quiz. We employed a repeated measures analysis to measure changes in knowledge after students watched the psychoeducational boredom video. Our findings reveal that students became more knowledgeable about boredom, learned something novel, and were interested in the intervention. The results are discussed in terms of the implications for research, theory, and practice.

## 1. Introduction

For many, postsecondary education is considered a place where students are engaged through intellectual and social stimulation, but this is not always the reality [1]. College and university students are susceptible to classroom boredom [2], which can negatively impact cognitive, motivational, and performance outcomes [3,4,5]. For example, boredom can interrupt learning by shifting attention away from the task at hand and promoting shallow learning strategies [6]. In a meta-analytic review, Tze and colleagues found that boredom was negatively related to varying motivation outcomes and that classroom boredom was most particularly detrimental to academic outcomes [5].

These patterns may be even more pronounced as students continue to manage online learning during the COVID-19 pandemic [7]. Some recent evidence revealed that when asked about the required transition to online courses, undergraduate students reported that the courses became less interesting, less enjoyable, and resulted in them paying less attention, among other negative outcomes [8]. Although the literature on boredom during the COVID-19 pandemic in postsecondary settings is still developing, research on students ranging from primary school to college levels, who were forced to homeschool as a result of the pandemic, found that students who were highly prone to boredom perceived their homeschooling to be more challenging, which subsequently lowered their adherence to learning at home [9]. Thus, boredom likely has a negative impact on students’ learning in at-home and online learning settings [10], making it an important emotion to study during the COVID-19 pandemic, which has forced many students to learn remotely.

In addition, since boredom does not seem to be naturally eliminated in postsecondary contexts [11], more explicit interventional strategies may be required. Two theories of emotion—the control-value theory (CVT) of achievement emotions [4] and the component process model of emotions (CPM) [12]—form the basis for creating a multi-sequenced online boredom intervention that follows general protocols for cognitive-behavior therapy [13]. The results presented herein focus on only Phase I of the boredom intervention training (BIT), which tests the effectiveness of a psychoeducational video to increase students’ knowledge of boredom.

### 1.1. Theoretical Framework

Boredom is known by all—“everyone has experienced it” (p. 315) [14]. It can rear its head during a lecture, in the workplace, and even while at home. However, fewer people know, or understand, boredom as a scholarly construct. Learning about boredom as a construct, separate from one’s personal experience of it, might be a helpful first step in understanding and managing this familiar emotion.

CVT’s three-dimensional taxonomy posits that achievement emotions can be classified by their subjective valence (positive or negative), physiological activation (activating or deactivating) and the object focus (outcome or activity) [15]. Within this taxonomy, boredom is theorized as a negative, deactivating, and activity emotion with low arousal [1,6]. CVT posits that boredom is experienced depending on certain combinations of students’ control and value appraisals in their academic settings. For example, boredom occurs when students do not value or have a good grasp of their academic activities or tasks (low value and low perceived control). Boredom can also occur when students have low perceived control, regardless of how much they value the course. Finally, boredom can occur when students have high perceived control, but little value for the course [4,6].

Because control and value appraisals are considered essential features of CVT, it is an excellent framework to consider how cognition or “thinking” is related to explaining academic boredom and its impact on performance. A variety of studies highlight the relationships between control and value appraisals, boredom, and academic achievement with a CVT lens [16,17,18,19,20]. Tze et al. conducted analyses using the Trends in International Mathematics and Science Study (TIMSS) 2015 data to examine the mediation of emotions (enjoyment and boredom) in the relationship between control and value appraisals and math performance in 53 education systems [19]. When examining boredom, they found that it mediated the relationship between these appraisal paths and math performance in 25 elementary school education systems.

Tze and Li [21] conducted a separate study to evaluate the mediating role of emotions on TIMSS 2015 science performance in both elementary and secondary school students. Specifically, boredom mediated the relationship between perceived control and science performance in 15 out of 35 Grade 8 education systems and between perceived value and science performance in 16 systems. Empirical studies have also tested the CVT of achievement emotions in physical education and physical activity settings [22,23], evidencing that the foundations of the theory operate in other achievement settings. In addition to its cognitive roots, boredom has distinct affective and behavioral components as well.

CVT goes on to further conceptualize achievement emotions, including boredom, comprising several components, such as affective (unpleasant feeling), physiological (lower arousal), cognitive (mind wandering), and motivational (look for escape) components [24,25]. Tze and colleagues conducted a validation study in Canadian and Chinese samples, supporting a four-component structure of boredom in learning [26]. The four-factor model comprised affective, physiological, cognitive, and motivational components as well.

Multiple conceptual frameworks can help to illuminate solutions to a pressing problem [27], and thus we turn to the component process model (CPM) of emotions as an additional perspective [28,29]. The CPM considers multiple components of emotions, such as the interaction of cognitive appraisals, bodily/physiological responses, motivated action tendencies, facial and vocal expressions, and subjective feelings [30,31]. Each component ranges in function from the evaluation of events, regulating one’s body, directing behavior, and communication [28]. According to CPM, when students “feel” bored in class, they may also “behave” in ways (e.g., sigh, yawn, slouch) that reinforce their boredom. It is this type of scholarly perspective on boredom involving cognitive appraisals (thinking), affective experiences (feeling), and behavioral responses (behaving) that postsecondary students need to understand if they are going to better cope. Thus, in the present study, we employ a psychoeducational approach with the goal to help students better understand boredom.

### 1.2. Psychoeducational Approaches in Cognitive Behavioral Therapy

Many cognitive behavior therapy (CBT) treatments start with, or involve, a psychoeducational element, e.g., [32,33] because of its functionality to teach skills and provide information [34]. Psychoeducational approaches are defined in terms of the accurate informing or educating of individuals who are pursuing help for psychosocial or mental wellbeing or specific diagnoses [35,36]. These approaches can vary greatly in format, including booklets, online programs, audiotapes, and videos. They are commonly incorporated in clinical settings to help to provide patients with information about symptoms, treatment and resources, and coping strategies for psychological or physical problems [37]. There are a number of psychoeducational approaches used in educational settings, for example, to change help-seeking strategies for mental health issues [38], to help students exhibiting depressive symptoms to manage stress [39], and even to show students how they can “grow their brain” in the context of encouraging adaptive mindsets (e.g., “Brainology”) [40]. Furthermore, psychoeducational interventions have been shown to reduce stress [41], suggesting the efficacy of this intervention approach. Given that boredom, similarly to stress, involves a non-clinical diagnosis and is commonly reported by students in learning contexts, the effectiveness of stress-based psychoeducational interventions provides some preliminary support that these approaches might be beneficial for boredom.

With the goal to help students manage their boredom, we combined CVT and CPM with a cognitive-behavioral method, e.g., [42,43] to create the first boredom-specific intervention, known as boredom intervention training (BIT). We chose CBT because research has shown its effectiveness in ameliorating negative emotions (e.g., anxiety, hopelessness, distress) [44,45]. It is also successful in helping individuals to identify problematic thought patterns that lead to feeling bored and to change behaviors that are reinforcing the problem [46]. The full intervention will comprise five phases (a) psychoeducation on boredom, (b) targeting misbeliefs and cognitions related to control and value appraisals, (c) guided imagery exposure and cognitive practice, (d) learning behavioral skills (e) and identifying boredom triggers to prevent relapse.

### 1.3. The Current Study

**Figure 1:**
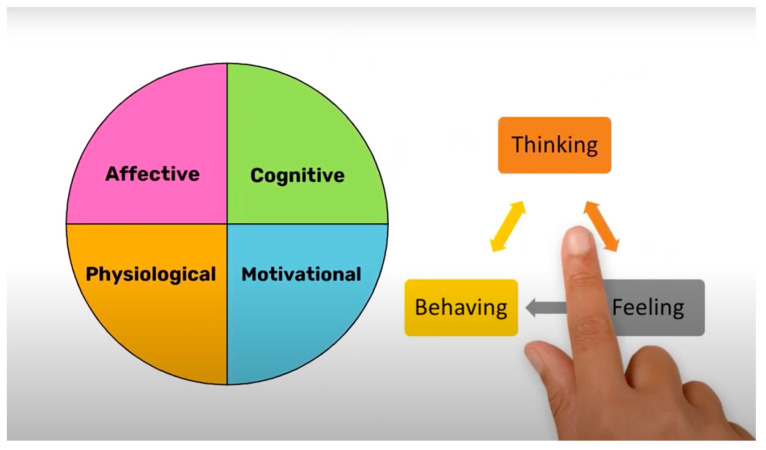
Thinking-feeling-behaving sequence of boredom. Note. The illustration is a screenshot from the boredom intervention training (BIT) video emphasizing the multidimensional components of boredom and the “thinking–feeling–behaving” sequence that triggers and maintains boredom.

The present study utilizes a novel application of a psychoeducational boredom video from a CBT perspective. We chose this method since broadly psychoeducational interventions are cost-effective and can be easily implemented [47]. Thus, to help students to manage their boredom ‘bit by bit’, we combined CVT and CPM as complementary theoretical approaches integrated into a psychoeducational video for the first phase of a boredom-specific intervention known as boredom intervention training (BIT). Both theories cover the cognitive (CVT) and physiological-behavioral (CPM) dimensions of boredom experienced by students. In Phase I, the goal was to provide undergraduate university students with systematic and structured knowledge [48] on how thinking, feeling, and behaving are interwoven and can trigger and maintain boredom (Figure 1). The essence of psychoeducation as a first phase is that the better students know their psychological challenges, in this case boredom, the more likely it is that they can embark on an adaptive path toward reducing this negative emotion [13].

Thus, the purpose of this study was to identify boredom frequency within the sample, to test the effectiveness of the psychoeducational boredom video in teaching participants about boredom, conduct a fidelity check ensuring participants did complete the session, e.g., [49], and gather interest as a proof of concept for the design. In doing so, we expand the existing literature on boredom by empirically testing a phase of a novel boredom intervention that teaches students about the various components (affective, physiological, cognitive, and motivational) that comprise boredom from a multi-theoretical perspective (CVT and CPM).

The objectives of Phase I of BIT were to: (a) describe the baseline levels of university boredom in general and specific class-related boredom; (b) increase students’ knowledge about boredom as a scholarly construct; (c) check the fidelity of BIT Phase I participant adherence with a consolidation exercise; and (d) determine the confidence of the Phase I design as engaging and motivating for participants to return for future phases. Thus, in the present study, we primarily focused on if students became more knowledgeable about boredom and if their interest in the intervention was sustained, not whether or not they experienced less boredom, which will be tested in a later phase. We hypothesized that students would indicate more accurate knowledge about boredom after watching the psychoeducational video.

## 2. Method

### 2.1. Participants and Procedure

Undergraduate students (*N* = 85) from a Canadian university were recruited to partake in the first session of the BIT program via an online advertisement called “Student Digest”, which is a weekly email about upcoming events, deadlines, and research opportunities. Of the sample, 70% were women, 26.7% men, and 3.3% were non-binary, and the participants ranged in age from 18 to 42 (*M* = 21.88). Among these students, 13% were in their first year, 17% in their second year, 27% in their third year, 38% in their fourth year, and 5% in their fifth year or higher of university. Regarding academic programs, 33% indicated they were in Science, 17% in Engineering, 17% in Arts, 7% in Medicine/Dentistry, 7% in Business, 7% in Agriculture, 5% in Law, and the remaining 7% were spread across Education, Health, and Kinesiology. See Table 1 for a summary of the study variables.

The recruitment post contained a link to a Google site that hosted Phase I of the BIT intervention. The first page of the site informed students about the study and that by clicking the “next” button (implied by overt action), they were consenting to participate in the study. Those who did not wish to participate could close the Internet window. Participants worked through four steps: (a) a baseline survey and knowledge quiz; (b) the psychoeducational boredom video; (c) a consolidation exercise as a fidelity check; and (d) a follow-up knowledge quiz.

The baseline survey comprised questions on information about students’ experience of boredom. The baseline and follow-up knowledge quizzes tested knowledge about boredom as a scholarly construct that should have been acquired through the boredom video. The boredom video was 2 min long and made with whiteboard animation to sustain attention. Immediately after participants viewed the psychoeducational boredom video, as a fidelity check, they completed the consolidation exercise that involved matching six video facts with corresponding video images. Finally, participants answered questions on their interest in the session. Our study had ethical approval from the researchers’ ethics review board.

### 2.2. Measures

Baseline Boredom. We assessed students’ baseline classroom-related and university boredom in general. We used four items for classroom-related boredom on a scale from 1 (strongly disagree) to 5 (strongly agree) from Bieleke et al.’s short version of the Achievement Emotion Questionnaire (AEQ-S, Cronbach’s α = 0.93) [50]. For these items, students were instructed to think about a specific class during the course of their studies when responding to the questions. Additionally, participants responded to a 1-item measure of general boredom frequency in university on a scale from 1 (never) to 10 (all the time) to rate how frequently they felt bored in university.

Psychoeducational Video and Fidelity Check. The psychoeducational boredom video was designed to provide students with systematic and structured knowledge [48] on how thinking, feeling, and behaviors are connected and can trigger and maintain boredom. This knowledge content was based on both emotion theories, CVT and CPM [4,28,29], that posit boredom has affective, cognitive, physiological, and behavioral dimensions and there is more to the emotion than just “feeling bored”. The video depicted scenarios in which postsecondary students feel bored (e.g., it began by portraying a student sitting slouched in class listening to another boring lecture thinking about all the things they are not getting done while their monotonous instructor drones on). The video narration discussed some empirical-based facts about boredom in the classroom and common misbeliefs. Following the video, participants completed a six-item matching activity for the purposes of consolidating the content presented in the video and providing a self-report fidelity check that participants viewed the psychoeducational boredom video.

Knowledge about Boredom. Students answered five knowledge-based multiple-choice questions about boredom in the baseline and follow-up quizzes. The questions asked students about: (a) their knowledge on the prevalence of boredom in university (Domain 1: Boredom experience), (b) the impact of boredom on learning (Domain 2: Impact of boredom), (c) the multidimensional complexity of boredom (Domain 3: Structure of boredom), (d) their knowledge on how boredom is maintained (Domain 4: Mechanism maintaining boredom), and (e) coping with boredom (Domain 5: Option to deal with boredom). The questions tested knowledge taught in the psychoeducational video and were coded 1 for correct responses and 0 for incorrect responses. Correct responses were summed such that participants could score a maximum of five and a minimum of zero on each quiz.

Learning and Interest. Participants indicated if they learned something new and were interested in future BIT sessions (dichotomous yes/no).

## 3. Results

### 3.1. Rationale for Analyses

We used a combination of frequencies and descriptive statistics to describe participants’ boredom and overall response to the Phase I study. We conducted a repeated measures analysis controlling for baseline classroom-related boredom to measure changes in knowledge after watching the video. In our repeated measure analysis, we opted to control for baseline boredom, since students experiencing higher boredom may have a unique or enhanced understanding of boredom. All of the analyses were conducted using SPSS.

### 3.2. Baseline Boredom

**Figure 2:**
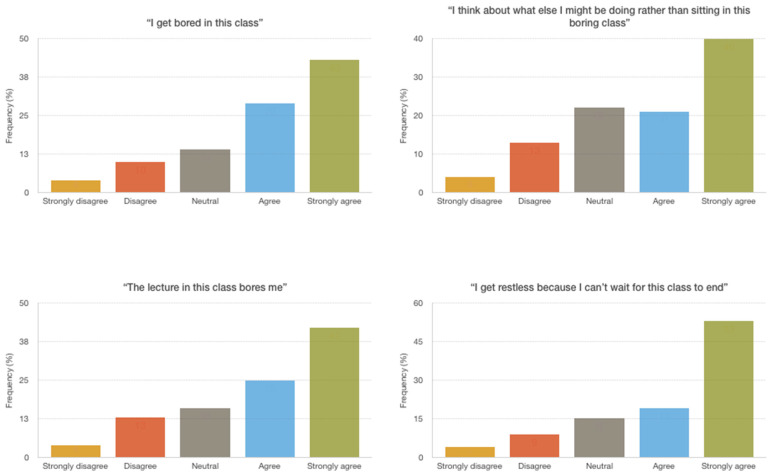
Student frequencies of reported classroom boredom at baseline. Note. Participants’ responses to classroom boredom items are displayed (prior to watching the psychoeducational boredom video).

On average, participants gave a rating of 6.41 on a 10-point scale for how frequently they experienced boredom in university in general. The majority of the students agreed to strongly agreed that they get bored in this class (73%); that the lecture in this class bores them (68%); that they think about what else they could be doing rather than sitting in the boring class (61%); and that they get restless because they cannot wait for the class to end (73%; see Figure 2 for item frequencies).

### 3.3. Consolidation Exercise (Fidelity Check)

On average, students attempted the matching consolidation exercise only once (95% response rate), with a mean of 5.9 out of 6 matches for their lowest score (94% response rate) and 5.9 out of 6 matches for their highest score (95% response rate). The close to perfect match on both the mean low and high scores suggests the high fidelity of implementation with participants attending to the video [49,51,52,53].

### 3.4. Knowledge about Boredom

Viewing the psychoeducational boredom video had a statistically significant effect on participants’ boredom knowledge, *F*(1, 77) = 5.64, *p* = 0.020, *MS* = 0.84, partial η^2^ = 0.07 (medium effect size according to conventions) [54], after controlling for baseline classroom-related boredom (see Table 2). Students’ scores for questions about boredom increased from an average of 3.1 out of 5 prior to viewing the video to an average of 4.5 out of 5 after the video. Prior to the video, the participant percentage obtaining correct answers ranged from 26% to 85% across the five items, suggesting that no participant had a fully accurate understanding of boredom as a scholarly construct prior to the psychoeducational video. Following the video, participants’ correct answers ranged from 73% to 99% (see Table 3 for items and frequencies).

### 3.5. Learning and Interest

In line with the results of the knowledge quiz, 93% of participants reported that they learned something new at the conclusion of the session. Moreover, 88% of participants indicated if there were more sessions, they would be interested to return.

## 4. Discussion

The main objective of Phase I of BIT was to increase students’ knowledge about boredom, and our findings reveal that, overall, it was effective. Most directly, based on change scores across two quizzes taken immediately before and after the psychoeducational video, students answered more knowledge-based questions correctly. Our results also suggest that participants were indeed experiencing boredom, that they attended to the video in a way indicative of high fidelity, and that they expressed interest in further sessions on boredom.

When considering students’ quiz scores prior to watching the psychoeducational boredom video, it is evident that there is knowledge to be learned, since the average overall quiz mean score was 60%. This finding suggests that students had some accurate knowledge about boredom, but that they also had misunderstandings (i.e., selected wrong answers). Such wrong answers may highlight that students’ subjective experiences or evaluations of boredom may not completely align with their objective understanding of the construct. Scherer noted that objective characteristics of an emotion can be assessed according to a person’s values, goals, and potential to cope with the emotion [31]. Furthermore, although the quiz items were carefully and intentionally crafted by the researchers to reflect accurate boredom knowledge, this can be a difficult task and some originally correct answers may have been guesses. For example, prior to viewing the psychoeducational video, 85% of students accurately answered the true or false item: “Boredom is as bad for your grades as test anxiety”. Conversely, only 26% of students accurately answered the multiple-choice item: “Approximately how many university students experience boredom during their classes?”. It can be assumed that more students would correctly guess a true or false item, with 50% chance of getting it right, than an item with a 25% chance.

Aligning with other CBT studies that use psychoeducational approaches, e.g., [55], it appears that teaching students about boredom can be an effective approach. At the end of the session, 93% of participants overall indicated that they had learned something new about boredom (subjective confirmation of new knowledge learned). Moreover, given that boredom is a familiar construct—indeed, the majority of participants indicated they were actively experiencing classroom-related boredom—our results suggest that the psychoeducational video helped participants to gain a better understanding of the empirical perspectives on boredom rather than their own experiences of the emotion. The psychoeducational video provides this knowledge base for students to anchor and explore their prior boredom experience. These findings resulted when controlling for baseline levels of boredom, implying that the psychoeducational boredom video is helpful for students regardless of how much boredom they are currently experiencing. Accurate knowledge of a phenomenon is a critical first step in cognitive-behavioral interventions [13,56] and the results suggest that Phase I of BIT provides a strong foundation for the remaining phases of the intervention.

Following the psychoeducational boredom video, students completed the matching consolidation exercise, designed as a fidelity check, with ease. This helps to provide support that participants viewed the psychoeducational video and were paying attention. Additionally, Phase I of BIT increased students’ interest in the larger intervention, with 88% of students indicating a desire for more sessions. We intentionally designed the animated video and consolidation exercise in an engaging way to provide students psychoeducation about boredom as well as build interest to return for future sessions. Our findings confirm that students learned something novel and were engaged enough to want to return to learn more about coping with boredom. Notably, interest was also captured at the recruitment stage of the study. After the recruitment advertisement was made public, 85 participants signed up for the study in under an hour, with over 20 additional individuals contacting the researchers, requesting to participate after the survey was closed. There appears to be an interest in learning about boredom, or at least interest in partaking in a study pertaining to that particular emotion.

### 4.1. Implications for Research, Theory, Practice

The research presented herein has important implications for intervention research, using theory to guide the development of interventions, and for practice. This study represents the first phase of the boredom intervention training program, which has been designed such that each phase can be evaluated individually, as is reported here for Phase I. As acknowledged, Phase I can equip learners with systematic and structured knowledge [48] on how thinking, feeling, and behaviors are interwoven in triggering and maintaining boredom. Phase I can help learners to identify the different components that give rise to boredom in whatever learning situation they are in (e.g., in class, online, during a presentation, etc.). Phase II will assist students in their academic settings to consider modifying certain beliefs about boredom (e.g., dysfunctional assumptions, negative and unrealistic views) [57,58], helping them to cognitively restructure their beliefs about boredom. Phases III and IV are intended to provide individuals with the cognitive and behavioral skills to manage their boredom based on Nett et al.’s recommendations [59]. Phase V deals with identifying triggers for relapse. Research must occur on each phase separately to ensure that they meet the intended objective and to build confidence that the intervention in its entirety stands the greatest chance to effectively reduce boredom. Furthermore, the full BIT will be designed with the purpose of reducing university boredom through these five sequential phases, and subsequently fostering learning-related motivation and achievement that is evidenced to be hindered by high boredom [6,15].

The boredom intervention draws on two theoretical frameworks: CVT and CPM. In Phase I, these frameworks guided the creation of the content for the psychoeducational boredom video. The video alludes to specific principles of CVT that can elicit boredom such as students’ control and value appraisals [20]. For example, the video addresses the fact that students can perceive boredom as being caused by the classroom environment—such as a monotonous instructor—a factor not under students’ control, or it can be caused by the person—such as if they do not hold value for the course or if they opt for distractions (e.g., social media). Furthermore, the video integrates Scherer’s CPM approach by describing to students the multidimensional nature of boredom, identifying the affective, cognitive, physiological, and motivational components [31]. An example of a student experiencing these various components of boredom in the classroom is briefly “walked through” to help guide students to link the theory to a real-life, and likely very relatable, example. Theoretical integration will be important for the development of later phases as well as to guide the selection of skills and strategies to deal with the multiple-component nature of boredom effectively.

Finally, there are some notable implications of the BIT program, even just this first phase, for practice. First, we have shown that students have misunderstandings about boredom that can be corrected through a psychoeducational video. The video itself could become a product to be integrated into undergraduate courses simply for the purposes of education. The full BIT intervention has possible instrumental value for equipping students with the necessary skills required to reduce the triggers of boredom. For example, these skills may help students to enhance their academic learning by removing interference with cognitive resources (e.g., attention processes in their learning tasks) that can be associated with boredom [6,15]. Such skills also have the potential to be transferable to other important areas in students’ lives, such as staying alert in mundane job tasks or volunteer activities, and even managing the emotion during boring team practices or training routines in sport settings. The product of this intervention has the potential to be used and shared widely across universities as an interactive and engaging way for students and instructors to combat student boredom and promote academic motivation and performance.

### 4.2. Limitations and Directions for Future Research

The results of this study need to be considered in light of the following limitations. First, we had a non-random treatment design and lack of a control group because we were interested in the effectiveness of the psychoeducation video to increase students’ knowledge. Second, although the session was not intended to reduce the experience of boredom, there may be advantages to assessing the effectiveness of each Phase of BIT in reducing the actual experience of boredom. However, we did not collect these data for the current study. Third, post-video consolidation data were collected from participants immediately after viewing the video. Future assessments would benefit from collecting data over a longer period of time to evidence sustained long-term effects of knowledge gained.

Finally, the data were collected during remote instruction required by public health restrictions during the COVID-19 pandemic, and so students may have been dealing with different sources of boredom as well as additional stressors. For example, recent research during the pandemic found that individuals with higher levels of boredom found compliance measures, such as social distancing, more challenging, which lead to lower adherence [60]. Notably, this could also indicate that students who are experiencing boredom are likely facing multiple boredom experiences in the classroom and in their day-to-day managing of the pandemic. Nonetheless, the gains in knowledge and interest in future sessions provides a strong foundation on which to continue building BIT.

### 4.3. Conclusions

In sum, our findings reveal that students in a Canadian university are currently experiencing high levels of boredom in specific classes and in university more generally. Our study showed that Phase I of a boredom intervention training program was effective in increasing students’ knowledge about boredom as a scholarly construct. Furthermore, the participants indicated adherence to the psychoeducational video in a way that reflected high fidelity, and encouragingly, they expressed interest in returning for future sessions to learn about boredom.

## Figures and Tables

**Table 1:** Summary of the study variables.

Measures	Items	Anchors	α	*M/%*	*SD*	Actual Range
Gender	1	Man		26.7%	–	–
		Woman		70%		
		Non-binary		3.3%		
Age	1	–		21.88	3.89	18–42
Ethnicity	1	Asian		41.1		
		Black		1.7%		
		Asian/White		1.7%		
		Caribbean		1.7%		
		Jewish		1.7%		
		Latin		1.7%		
		Middle Eastern		1.7%		
		Indian/White		1.7%		
		South Asian		1.7%		
		South East Asian		3.4%		
		West Indian		1.7%		
		White/Caucasian		39.7%		
Year of university	1	First		13.3%		
		Second		16.7%		
		Third		26.7%		
		Fourth		38.3%		
		Fifth or higher		5.0%		
Course format	1	Asynchronous		26.3%		
		Synchronous		28.7%		
		Blended		25.0%		
		Face-to-face		20.0%		
Classroom-related boredom	4	1 = strongly disagree	0.93	15.82	4.32	5–20
		5 = strongly agree				
University boredom	1	1 = never	–	6.41	1.87	2–10
		10 = all the time				
Baseline boredom knowledge	5	–	–	3.08	1.07	0–5
Follow-up boredom knowledge	5	–	–	4.44	0.73	2–5
Consolidation exercise:						
Attempts	1	–	–	1.02	0.16	1–2
Highest score	1	–	–	5.89	0.55	2–6
Lowest score	1	–	–	5.91	0.45	3–6

Note. Students reported being in the following faculties: Agricultural, Life, and Environmental Sciences, Arts, Business, Education, Health, Engineering, Kinesiology, Law, Medicine and Science.

**Table 2:** Analysis of Phase I boredom knowledge.

Test of Within-Subject Contrasts					
	*df*	Mean Square	*F*	*p*	Partial *η*^2^
Baseline and follow-up boredom knowledge	1	0.84	5.64 *	0.020	0.068
Baseline and follow-up boredom knowledge x baseline boredom	1	<0.01	0.025	0.874	0.000
Error	77	0.15			

Note. * *p* < 0.05.

**Table 3:** Knowledge-based multiple-choice domains, frequencies, and mean.

Domain	Time 1 (% Correct)	Time 2 (% Correct)
Boredom experience	26%	81%
Impact of boredom	85%	99%
Structure of boredom	79%	94%
Mechanism maintaining boredom	71%	99%
Option to deal with boredom	45%	73%
Overall *M* (*SD*)	3.08 (1.07)	4.44 (0.73)

Note. Multiple choice items can be requested from the corresponding author.

## Data Availability

Students in our study did not consent to the data being made publicly available.
